# Novel *Wx* Gene Functional Markers for High-Resistant Starch Rice Breeding

**DOI:** 10.3390/genes17010089

**Published:** 2026-01-14

**Authors:** Jie Ouyang, Zichao Zhu, Yusheng Guan, Qianlong Huang, Tao Huang, Shun Zang, Chuxiang Pan

**Affiliations:** 1 Chongqing Academy of Agricultural Sciences, Chongqing 401329, China; zhuzichao@cqaas.cn (Z.Z.); guanyusheng502@163.com (Y.G.); hql1987414@163.com (Q.H.); 2 Dianjiang County Agricultural Technology Promotion Station, Chongqing 408300, China; insistence0906@sina.com; 3 Chongqing Seed Station, Chongqing 401121, China; 19923352579@163.com; 4 Hunan Shennong Dafeng Seed Industry Technology Co., Ltd., Changsha 410200, China; 18807402538@139.com

**Keywords:** rice, resistant starch, *Wx* gene, tetra-primer ARMS-PCR, functional marker

## Abstract

**Background/Objectives**: Chemical methods for quantifying resistant starch (RS) in rice are labor-intensive, costly, and lack high repeatability, creating a bottleneck in breeding. This study aimed to develop specific, codominant molecular markers for the *Wx* gene to enable rapid and accurate genotype screening for RS content, thereby accelerating the development of high-RS rice varieties. **Methods**: Based on sequence alignment of the *Wx* gene in rice varieties with divergent RS content, a key single-nucleotide polymorphism was targeted. Two sets of tetra-primer amplification refractory mutation system polymerase chain reaction (ARMS-PCR) markers, T-*Wx*9-RS1 and T-*Wx*9-RS2, were designed. These markers were used to genotype diverse rice varieties and F_4_ segregating populations, with results validated against standard chemical assays. **Results**: Sequence analysis identified a critical T → C base mutation at position 202 of the ninth exon in high-RS varieties. The developed ARMS-PCR markers successfully and consistently distinguished all three possible genotypes (homozygous mutant, homozygous wild-type, and heterozygous). The genotyping results showed complete concordance with the phenotypes determined by chemical methods. **Conclusions**: The developed molecular markers, T-*Wx*9-RS1 and T-*Wx*9-RS2, provide a rapid, reliable, and cost-effective tool for marker-assisted selection of high resistant starch content in rice. Their implementation can significantly enhance screening efficiency and expedite the breeding pipeline for novel, nutritionally improved rice cultivars.

## 1. Introduction

Rice (*Oryza sativa* L.), a major global food crop, not only is a vital source of energy for humans but also holds significant cultural importance [1]. As health awareness has increased, the focus on rice quality has expanded from traditional yield and taste to include nutritional and health benefits. High-resistance starch (RS) rice, known for its unique physiological functions that help reduce post-meal blood sugar spikes and lower the risk of diseases such as type II diabetes and obesity, is becoming a key target in the breeding of functional rice [1,2]. Resistant starch, a component of starch that is difficult to digest in the small intestine, ferments in the gut to produce short-chain fatty acids, which are beneficial to gut health [1,2].

The starch in the rice endosperm is primarily composed of amylose and amylopectin, and its physicochemical properties affect the processing, taste, and nutritional quality of the rice and, especially, the content of RS. Granule-bound starch synthase I (GBSSI), encoded by the *Wx* gene, is a key enzyme that regulates the amylose content (AC) in the rice endosperm [3,4]. Numerous studies have shown that subtle changes in the expression levels and activity of the *Wx* gene can considerably alter the amylose content, which in turn affects the gelatinization temperature (GT), viscosity profile (RVA profile), eating and cooking quality (ECQ), and potential formation of RS [3,4,5,6]. Therefore, the *Wx* gene is considered a core regulatory module for improving the taste and nutritional quality of rice, particularly the content of RS [3].

Precisely regulating the expression of the *Wx* gene is an effective strategy for enhancing rice quality. Traditional breeding methods regulate the amylose content by selecting and combining different natural alleles (such as *Wx^a^* and *Wx^b^*). Molecular biology research has further revealed the complexity of *Wx* gene expression regulation, including cis-acting elements in the promoter region (such as CAAT-box and TATA-box sequences), structural changes in the 5′-UTR region [3], and the regulatory mechanisms of upstream transcription factors such as OsEBP89, OsSPL14, and OsGSK5/OsSK41 on *Wx* [7,8]. These findings lay the foundation for a deeper understanding of the molecular network involved in amylose formation.

In recent years, gene editing technologies, particularly the CRISPR/Cas9 system, have provided powerful tools for achieving precise targeting and modification of the *Wx* gene. Research has shown that by editing the *Wx* gene promoter [3,9] or coding region [5], a variety of new rice materials with different amylose contents and physicochemical properties (such as the gelatinization temperature and resistant starch content) can be created [3,5,6,8,9]. For example, editing the *Wx* promoter (such as reducing the expression of *Wx^a^*) can reduce chalkiness and improve appearance quality [9]; creating specific *Wx* alleles or combining them with other starch synthesis-related genes (such as SBEIIb, SSIIa, and FLO2) can result in rice materials with high amylose content and high-resistant starch characteristics [10,11,12]. These successful cases highlight the core role of the *Wx* gene locus in the breeding of rice with specific qualities, including high RS.

To more effectively apply these *Wx* gene-based improvements in breeding practices, the development of closely linked functional molecular markers is crucial. Functional markers (FMs) are molecular markers developed from allele variants that cause phenotypic variations in genes or regulatory regions, such as SNPs and Indels. Compared with conventional markers, these markers significantly increase the precision and efficiency of marker-assisted selection (MAS). In crop breeding, functional markers are widely recognized as key tools for accelerating the integration of target traits, such as disease resistance and quality traits [13,14,15,16,17,18,19,20,21,22]. In the context of rice-resistant starch breeding, developing high-precision functional molecular markers at key functional sites of the *Wx* gene (including its promoter, 5′-UTR, and coding region) can accurately distinguish different alleles, predict amylose content and related starch characteristics (such as RS potential), and enable rapid and precise tracking and aggregation of superior alleles in breeding populations, thus significantly shortening the breeding cycle and improving selection efficiency [13,19,20].

In summary, the *Wx* gene is the core switch regulating the amylose content and physicochemical properties of the rice endosperm, and its diversity is the key genetic basis for differences in rice quality, including RS content [3,5,6]. A deep study of the regulatory mechanisms of the *Wx* gene and the development of new functional molecular markers based on key functional sites will provide strong technical support for the efficient breeding of new rice varieties with excellent taste qualities and high RS contents [1,2,9,11,13]. This study aims to introduce methods for the development of new functional molecular markers around the *Wx* gene locus, their validation, and their practical application in the breeding of high-RS rice.

## 2. Materialsand Methods

### 2.1. Plant Materials

Fourteen rice varieties and seven F4 populations were provided by the Rice Research Institute of the Chongqing Academy of Agricultural Sciences. The rice varieties were Li, Lan, HR52, QR, ML, BR50, BR19, CN-9, Sx, BR18, Ky43, CN-5, BR89, and BR28, and the F4 hybrid populations were Lan/CN-5, Lan/CN-9, Lan/Sx, QR/Sx, QR/Ky43, Li/BR50, and QR/BR50. The seeds were sown in a seedling bed after disinfection and germination. The 25-day-old seedlings were then transplanted into the Dianjiang experimental field (Chongqing, China, 30°25′31″ N 107°24′1″ E, with a typical subtropical monsoon climate and purplish clay soil) of the Rice Research Institute of Chongqing Academy of Agricultural Sciences. Field management followed local high-yielding practices: transplanting at a spacing of 20 cm × 26.7 cm with two seedlings per hill, applying fertilizer at the rate of 150 kg N/ha, 75 kg P_2_O_5_/ha, and 100 kg K_2_O/ha, and maintaining a shallow water layer except during the mid-season drainage period for about 10 days to control ineffective tillering.

### 2.2. Determination of Apparent Amylose and RS Contents

The apparent amylose content (AAC) was determined according to the Chinese National Agricultural Industry Standard NY/T2639-2014. The four AAC standard samples (1.2, 11.2, 16.8 and 26.8%) were provided by the Rice Product Quality Inspection and Supervision Testing Center of the China National Rice Research Institute of the Ministry of Agriculture. In rice, AAC can be classified into the following grades: waxy (0–2%), very low (5–12%), low (12–20%), medium (20–25%), or high (25–33%) [23]. The RS content was determined according to the ministry standard NY/T2638-2014 and calculated using the following formula: RS (%) = Sample/Standard glucose × 0.9 × 0.1 × 10.3/0.1 × 100/w (where w represents the dry weight of the sample in mg). Each measurement was performed with three technical replicates. On the basis of previous studies and our own data, we classified the rice RS content into three grades: low (<3%), medium (3–6%), and high (>6%).

### 2.3. DNA Extraction from Rice Leaves

Fresh leaves of the different rice varieties and F4 populations were collected at the tillering stage, and genomic DNA was extracted via the CTAB method [24].

### 2.4. Sequence Comparison, Sequencing and Primer Design

The full-length DNA sequence of the *Wx* gene (chromosome 6, nc_029261.1 (1765524.1770644)) was downloaded from the NCBI database. The PCR primers used were designed with Primer3 (https://bioinfo.ut.ee/primer3-0.4.0/ (accessed on 27 May 2020). The genomic DNA of 14 rice varieties with different RS contents was amplified to create templates for the PCR products. The PCR products were sent to Sangon Biotech (Shanghai) Co., Ltd. (Shanghai, China), for sequencing. The sequencing results were compared with those of Vector NTI Advance 11.5.1 to determine the differences between high-RS- and low-RS-content rice varieties. Primers for tetra-primer amplification refractory mutation system polymerase chain reaction (ARMS-PCR) were designed on the basis of these differences and synthesized by Sangon Biotech (Shanghai) Co., Ltd., using the online four-primer analysis software PRIMER1 (http://primer1.soton.ac.uk/primer1.html (accessed on 11 June 2021) reported by Ye et al. [25].

### 2.5. PCR Amplification and Recovery of the Wx Allele Fragment

The *Wx* allele fragment was amplified using a Phanta Max Super-Fidelity DNA Polymerase Kit (P505-d1; Nanjing Vazyme Biotech Co., Ltd., Nanjing, China). The 50 μL PCR system included 2.0 μL of template DNA (20 ng/µL), 2.0 μL each of upstream and downstream primers (10 μM), 25.0 μL of 2× Phanta Max buffer, 1.0 μL of dNTP mixture (10 mM each), 1.0 μL of Phanta Max Super-Fidelity DNA Polymerase, and 17.0 μL of ddH2O. The PCR procedure was as follows: predenaturation at 95 °C for 3 min; 35 cycles of 95 °C for 15 s, 54 °C for 15 s, and 72 °C for 3 min; extension at 72 °C for 5 min; and cooling at 10 °C for 10 min. A bromophenol blue indicator was added to the amplification product for later use. The reaction product was electrophoresed on an agarose gel with 1.5% DuRed nucleic acid dye at 150 V for 30 min. The target band was removed on a UV transmission table and sent to Sangon Biotech (Shanghai) Co., Ltd., for sequencing.

### 2.6. Genotype Identification

The 20 μL PCR reaction mixture included 1.0 μL of template DNA (20 ng/µL), 0.5 μL of each of the four primers (10 μM), 10.0 μL of 2× 3G Taq Master Mix for PAGE (red dye) (P115-01, Nanjing Vazyme Biotech Co., Ltd.), and 7.0 μL of ddH2O. The PCR procedure was as follows: predenaturation at 95 °C for 3 min; 35 cycles of 95 °C for 15 s, 61 °C for 15 s, and 72 °C for 30 s; extension at 72 °C for 5 min; and cooling at 10 °C for 10 min. The PCR products were analyzed via 10% polyacrylamide gel electrophoresis to detect polymorphisms. Offspring with genotypes consistent with the low-RS parent were marked with “−”, those with genotypes consistent with the high-RS parent were marked with “+”, and those with heterozygous genotypes were marked with “H”.

## 3. Results

### 3.1. AAC and RS Contents

The AACs and RS contents of the 14 different rice varieties are shown in Table 1. The AACs of Li, QR, Lan and HR52 ranged from 14.86 to 15.67%, indicating low-amylose varieties, and the RS contents ranged from 0.44 to 0.57%, indicating low-RS varieties. The AACs of BR18, BR19, BR50, BR28, Sx, Ky43, BR89 and CN-5 ranged from 21.26 to 24.99%, indicating medium-amylose varieties, and the RS contents ranged from 6.25 to 9.10%, indicating high-RS varieties. The AACs of CN-9 and ML were 25.35 and 25.78%, respectively, indicating high-amylose varieties, and the RS contents were 10.66 and 6.68%, respectively, indicating high-RS varieties. Correlation analysis revealed a significant positive correlation between the RS content and AAC of each variety (r = 0.913, *p* ≤ 0.01).

### 3.2. Identification and Primer Design of Specific SNPs in the Wx Gene of High-RS Rice Varieties

**Figure 1:**
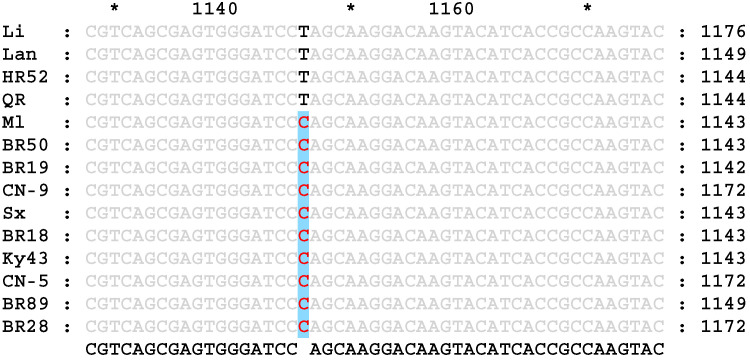
Partial nucleotide sequences of exon 9 of the *Wx* gene in different rice varieties. The gray letters represent the same nucleotide sequence, and the black and red letters indicate base mutations in T → C at position 202.

**Figure 2:**
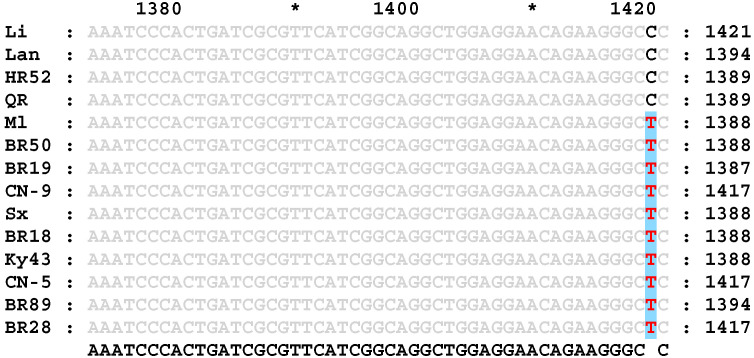
Partial nucleotide sequences of exon 10 of the *Wx* gene in different rice varieties. The gray letters represent identical nucleotide sequences, while the black and red letters indicate base mutations in C → T located at position 115.

The primers WL3 and WR (Table 2) were designed according to the rice *Wx* gene sequence in the NCBI database, and the genomic DNA of 14 rice varieties with different RS contents was amplified as a template. A 3043 bp fragment was obtained by sequencing. The sequence similarities and differences between the high-RS- and low-RS-content rice varieties were analyzed. Compared with that of low-RS-content rice, the *Wx* gene of high-RS-content rice was missing 4 bases; a T → C base mutation at position 202 of exon 9 (Figure 1) and a C → T mutation at position 115 of exon 10 (Figure 2) were detected in high-RS-content rice, in addition to several base mutations in introns.

Two sets of primers, T-*Wx*9-RS1 and T-*Wx*9-RS2 (Table 2), were designed using online software (http://primer1.soton.ac.uk/primer1.html (accessed on 11 June 2021)). These primers were designed to detect the base mutation at position 202 of exon 9 of the *Wx* gene in rice, which is closely related to high RS content.

### 3.3. Validation of Molecular Markers

**Figure 3:**
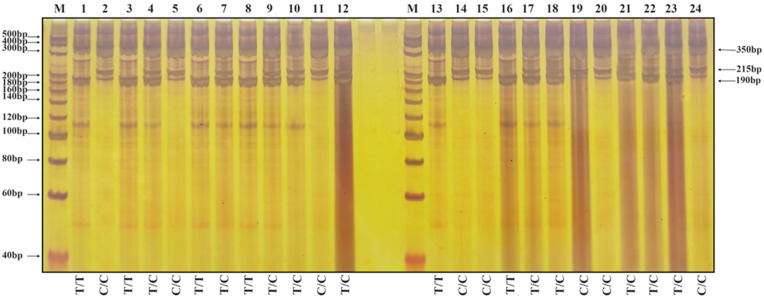
Nucleic acid electrophoresis results after amplification of the genomic DNA of F4 generation rice plants using the primer set T-*Wx*9-RS1. The expected PCR products are labeled on the right. Two bands of 350 and 190 bp indicate a low-RS-content rice variety, two bands of 350 and 215 bp indicate the homozygous genotype of a high-RS-content rice variety, whereas three bands of 350, 190 and 215 bp indicate the heterozygous genotype of a high-RS-content rice variety. Lane M: 20 bp DNA Ladder Marker from TaKaRa, Lane 1: Parent Li, Lane 2: Parent BR50, Lanes 3–12: F4 plants from cross Li/BR50 (7553 2-1 to 7553 2-10). Lane 13: Parent QR, Lane 14: Parent BR50, Lanes 15–24: F4 plants from cross QR/BR50 (7586 2-1 to 7586 2-10).

**Figure 4:**
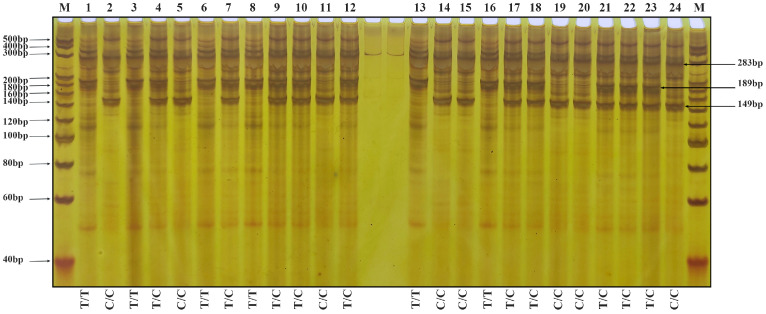
Nucleic acid electrophoresis results after amplification of the genomic DNA of F4 generation rice plants using the primer set T-*Wx*9-RS2. The expected PCR products are labeled on the right. Two bands of 283 and 189 bp indicate a low-RS-content rice variety, two bands of 283 and 149 bp indicate the homozygous genotype of a high-RS-content rice variety, whereas three bands of 283, 189 and 149 bp indicate the heterozygous genotype of a high-RS-content rice variety. Lane M: 20 bp DNA Ladder Marker from TaKaRa, Lane 1: Parent Li, Lane 2: Parent BR50, Lanes 3–12: F4 plants from cross Li/BR50 (7553 2-1 to 7553 2-10). Lane 13: Parent QR, Lane 14: Parent BR50, Lanes 15–24: F4 plants from cross QR/BR50 (7586 2-1 to 7586 2-10).

The molecular markers of 40 individual plants in the F4 population were verified. The results of nucleotide electrophoresis of representative plants are shown in Figure 3 and Figure 4. The results of amplification using the primer group T-*Wx*9-RS1 are shown in Figure 3. Two bands of 350 and 190 bp indicate a low-RS-content rice variety; two bands of 350 and 215 bp indicate the homozygous genotype of a high-RS-content rice variety (T → C base mutation at position 202 of the ninth exon of the *Wx* gene in both strands), whereas three bands of 350, 190 and 215 bp indicate the heterozygous genotype of a high-RS-content rice variety (T → C base mutation in one strand).

The results of amplification using the primer group T-*Wx*9-RS2 are shown in Figure 4. Two bands of 283 and 189 bp indicate a low-RS-content rice variety; two bands of 283 and 149 bp indicate the homozygous genotype of a high-RS-content rice variety (mutation in both strands), whereas three bands of 283, 189 and 149 bp indicate the heterozygous genotype of a high-RS-content rice variety (mutation in one strand). Individual plants from the F4 generation were analyzed with the two primer sets, and the results were consistent.

**Figure 5:**
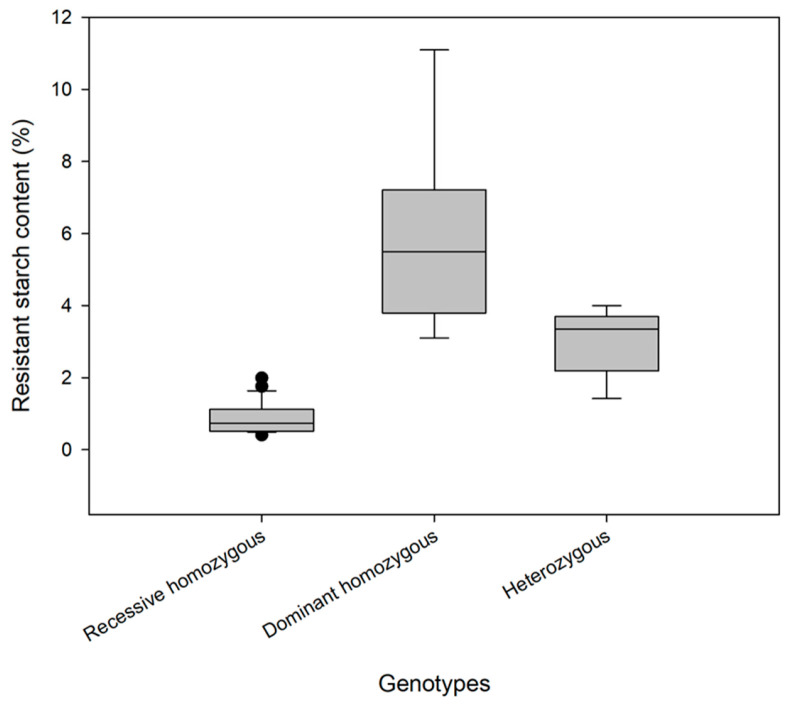
Box plot of resistant starch content in 40 individual plants of different genotypes.

The genotypes and RS contents of 40 individual plants are shown in Table 3 and Figure 5. Among the 40 F4 generation plants, 23 genotypes were consistent with those of the low–RS-content parent (without the mutant *Wx* gene, indicated by the symbol “−”), and the RS content was 0.41–1.99%, with an average of 0.88%. The genotypes of 8 plants were consistent with those of the high–RS-content parent (homozygous for the mutant *Wx* gene, indicated by the symbol “+”), and the RS content was 3.10–11.10%, with an average of 5.91%. The genotypes of 9 plants were heterozygous (inherited the mutant *Wx* gene from one parent, indicated by the letter “H”), and the RS content was 1.42–4.00%, with an average of 3.00%. These findings indicate that the RS content of a rice variety can be accurately predicted according to its genotype.

## 4. Discussion

### 4.1. Core Findings and Marker Utility

This study identified a novel, synonymous T → C SNP within exon 9 of the rice *Wx* gene that exhibits a perfect diagnostic association with elevated resistant starch (RS) content across our biparental populations. The key achievement is the successful development and validation of two robust, codominant tetra-primer ARMS-PCR markers (T-*Wx*9-RS1 and T-*Wx*9-RS2) precisely targeting this SNP. These markers efficiently discriminated among all three possible genotypes (homozygous for the low-RS allele, homozygous for the high-RS allele, and heterozygous) within parental lines and F_4_ segregating populations. The observed complete concordance between marker genotypes and chemically quantified RS levels (Table 3, Figure 5) underscores their immediate, high practical value for marker-assisted selection (MAS). This breakthrough equips breeders with a cost-effective, rapid, and accurate tool to screen for the high-RS trait in early generations, dramatically accelerating the breeding pipeline compared to reliance on time-consuming, cumbersome phenotypic assays alone.

### 4.2. Interpretation of the Synonymous Mutation and Its Potential Mechanisms

A central finding demanding careful interpretation is the robust association of a synonymous SNP with a distinct phenotypic effect. While our data unequivocally establishes this SNP as an outstanding molecular marker, its direct functional role remains elusive. We consider several non-exclusive possibilities: (1) Linkage Disequilibrium (LD): The T202C SNP likely resides in extremely tight LD with the true, yet unidentified causal variant. This causal mutation could be a cis-regulatory element influencing *Wx* expression levels or a non-synonymous change within another gene inside the haplotype block. (2) Effects on mRNA Processing: Synonymous mutations can profoundly impact mRNA splicing efficiency, stability, or secondary structure. The exon 9 SNP may disrupt an exonic splicing enhancer/silencer motif or alter the co-translational folding of the GBSSI protein, potentially compromising its activity or abundance. (3) Translational Kinetics: Altered codon usage, even for the identical amino acid, can modulate translation speed, thereby influencing protein folding and function.

We emphasize that our current experimental design—association analysis within a mapping population—cannot definitively distinguish between these scenarios. Therefore, we present the T202C SNP primarily as a highly reliable and tightly linked diagnostic marker for breeding applications. Definitive proof of causality necessitates future functional studies, such as in vitro splicing assays, allele-specific expression analysis, or the creation of isogenic lines differing solely at this SNP via precise gene editing.

### 4.3. Marker Development, Application, and Technical Considerations

The *Wx* locus stands as a well-documented major regulator of amylose and RS content. Our work introduces a novel, practical tool to the arsenal of *Wx*-based markers. Our deliberate choice of tetra-primer ARMS-PCR offered an optimal balance of accuracy, speed, and affordability, perfectly suited for routine breeding applications. However, we must acknowledge the technique’s inherent limitation: its success critically depends on specific primer design. Our inability to develop a functional ARMS-PCR marker for the exon 10 SNP, despite its established association with gel consistency [26], underscores this critical limitation. The probable cause is the complex genomic context surrounding that SNP, such as high sequence similarity to other regions or stable secondary structures, which prevented the design of four specific primers that meet all stringent requirements for robust amplification.

The two successful markers developed here precisely convert genotype data into a clear breeding decision: select plants harboring the homozygous “C” allele (or potentially heterozygotes for further advancement) to enrich for high RS content. This direct genotype-to-phenotype link, rigorously validated in our populations, forms the cornerstone of their practical value.

### 4.4. Limitations, Generalizability, and Future Perspectives

While our results are promising, several limitations must be noted to frame the appropriate scope of the markers’ application: (1) Genetic Background and Generalizability: Our validation occurred within specific crosses. The utility of these markers across the broad diversity of rice germplasm (e.g., indica vs. japonica, landraces vs. elite lines) hinges critically upon the conservation of the causal haplotype tagged by our SNP. We strongly recommend preliminary screening of target breeding panels to confirm the marker-trait association before large-scale deployment. (2) Environmental Effects and G × E: This study was conducted in a single environment. RS content, like many quality traits, is susceptible to environmental influences such as temperature during grain filling. The potential for genotype-by-environment (G × E) interaction remains unassessed. Therefore, implementing rigorous multi-location and multi-season trials constitutes an essential next step. These trials are crucial to evaluate the stability of the phenotype predicted by these markers and validate their effectiveness across diverse growing conditions. (3) Statistical Analysis: The statistical correlations presented, while clear and significant, are initial. Future studies utilizing larger, more diverse populations should employ more sophisticated models to account for additional genetic or environmental covariates.

In conclusion, the functional markers developed herein offer a rigorously validated, highly efficient tool for MAS in high-RS rice breeding within analogous genetic backgrounds. Future work must prioritize: (1) rigorous validation of these markers across independent and diverse germplasm collections; (2) expanding trials to multiple environments to critically assess stability; and (3) initiating fine-mapping or functional studies to definitively elucidate the precise biological mechanism underpinning the observed perfect association. In the breeding program, parallel selection using these markers alongside functional markers of key genes regulating starch quality, such as *SSIIa* and *SBEIIb*, will provide an efficient approach to achieve strategic accumulation of superior alleles and develop new materials with improved nutritional quality.

## 5. Conclusions

The results of the present study identified a significant association between the T → C base mutation at position 202 of exon 9 of the rice *Wx* gene and the formation of highly resistant starch, and the two sets of molecular markers, T-*Wx*9-RS1 and T-*Wx*9-RS2, which were developed for the base mutation at this position, can effectively distinguish among the three genotypes. Moreover, the genotypes are in complete agreement with the results of the chemical method. These two sets of molecular markers can be used to achieve rapid and accurate detection of resistant starch genotypes and accelerate the selection of new rice varieties with high starch resistance.

While the developed *Wx* gene functional markers show great promise in high-RS rice breeding, it is important to note that their performance may vary across different genetic backgrounds and environmental conditions. Future research should focus on validating these markers in diverse rice populations and under various field conditions, as well as optimizing breeding strategies to maintain high RS content while improving yield and disease resistance. Additionally, efforts should be made to simplify and reduce the cost of marker detection techniques to enhance their applicability in large-scale breeding programs.

## 6. Patents

Patent number: ZL 2022 1 0541772.0.

## Figures and Tables

**Table 1:** Amylose and RS contents of different rice varieties.

Variety	AAC (%)	AAC Grade	RS (%)	RS Grade
Li	14.86 ± 0.39 i	low	0.48 ± 0.14 g	low
QR	15.01 ± 0.28 hi	low	0.44 ± 0.22 g	low
Lan	15.14 ± 0.29 hi	low	0.48 ± 0.18 g	low
HR52	15.67 ± 0.17 h	low	0.57 ± 0.24 g	low
BR18	21.26 ± 0.42 g	medium	7.78 ± 0.42 de	high
BR19	22.68 ± 0.47 f	medium	8.33 ± 0.39 bcd	high
BR50	23.28 ± 0.53 ef	medium	9.10 ± 0.47 b	high
BR28	23.77 ± 0.29 de	medium	8.09 ± 0.68 cd	high
Sx	23.85 ± 0.25 de	medium	8.31 ± 0.62 bcd	high
Ky43	24.24 ± 0.33 cd	medium	6.25 ± 0.56 f	high
BR89	24.85 ± 0.76 bc	medium	8.94 ± 0.70 bc	high
CN-5	24.99 ± 0.38 b	medium	6.99 ± 0.53 ef	high
CN-9	25.35 ± 0.49 ab	high	10.66 ± 0.88 a	high
ML	25.78 ± 0.43 a	high	6.68 ± 0.68 f	high

Significant differences are indicated by different letters in the same column (*p* < 0.05). AAC, apparent amylose content; RS, resistant starch.

**Table 2:** Sequencing primers and related markers for detecting specific SNP variations in the rice *Wx* gene.

Marker Name	Primer Name	Primer Sequence
	WL3	GCAGATCAAGGTTGCAGACA
	WR	TGGCAATAAGCCACACACAT
T-*Wx*9-RS1	W9-IF1	GCATGGACGTGAGTACT
	W9-IR1	ACTTGGCGGTGGATGTACTTGTCCTTGATG
	W9-OF1	GGAGGAGGAAGATCAACTGGATGAA
	W9-OR1	TTGCCTGAAATTGTTACTCATTCTTGCC
T-*Wx*9-RS2	W9-IF1	GCATGGACGTGAGTACT
	W9-IR1	ACTTGGCGGTGGATGTACTTGTCCTTGATG
	W9-OF2	CCCGTACTACGCCGAGGAGCTCATCT
	W9-OR2	TGCCTGAAATTGTTCACTCATTCTTGCCTT

WL3 and WR are the upstream and downstream primers, respectively, that were used for sequencing.

**Table 3:** Genotypes and corresponding RS contents of 40 individual plants.

Number	Source	Genotype	Resistant Starch (g/100 g)	Number	Source	Genotype	Resistant Starch (g/100 g)
2021p1637	Lan/CN-5	−	0.50 ± 0.16	2021p1657	QR/Ky43	−	0.52 ± 0.21
2021p1638	Lan/CN-5	−	0.52 ± 0.09	2021p1658	QR/Ky43	−	0.59 ± 0.23
2021p1639	Lan/CN-5	−	0.49 ± 0.15	2021p1659	QR/Ky43	−	0.62 ± 0.14
2021p1640	Lan/CN-9	−	1.76 ± 0.24	2021p1660	Lan/CN-9	+	3.96 ± 1.66
2021p1641	Lan/CN-9	−	1.30 ± 0.35	2021p1661	Lan/CN-9	+	3.74 ± 1.65
2021p1642	Lan/Sx	−	1.31 ± 0.33	2021p1662	Li/BR50	+	4.52 ± 0.18
2021p1643	Lan/Sx	−	0.51 ± 0.24	2021p1663	QR/BR50	+	7.24 ± 0.94
2021p1644	QR/BR50	−	1.99 ± 0.65	2021p1664	QR/BR50	+	6.47 ± 0.96
2021p1645	QR/BR50	−	0.91 ± 0.10	2021p1665	QR/BR50	+	11.10 ± 1.70
2021p1646	QR/BR50	−	0.41 ± 0.04	2021p1666	QR/BR50	+	7.14 ± 1.38
2021p1647	QR/BR50	−	1.04 ± 0.07	2021p1667	QR/BR50	+	3.10 ± 0.44
2021p1648	QR/BR50	−	0.77 ± 0.17	2021p1668	Lan/Sx	H	4.00 ± 1.49
2021p1649	QR/BR50	−	0.78 ± 0.11	2021p1669	Lan/Sx	H	3.48 ± 1.19
2021p1650	QR/BR50	−	0.68 ± 0.10	2021p1670	QR/BR50	H	1.53 ± 0.45
2021p1651	QR/BR50	−	0.68 ± 0.14	2021p1671	QR/BR50	H	1.42 ± 0.40
2021p1652	QR/Sx	−	1.12 ± 0.18	2021p1672	QR/BR50	H	3.04 ± 0.73
2021p1653	QR/Sx	−	1.45 ± 0.44	2021p1673	QR/BR50	H	3.35 ± 0.33
2021p1654	QR/Sx	−	1.06 ± 0.17	2021p1674	QR/BR50	H	2.84 ± 0.56
2021p1655	QR/Ky43	−	0.73 ± 0.28	2021p1675	QR/BR50	H	3.91 ± 0.94
2021p1656	QR/Ky43	−	0.59 ± 0.18	2021p1676	QR/BR50	H	3.44 ± 0.96

Offspring with genotypes consistent with the low–RS-content parent are indicated by “−”, those with genotypes consistent with the high–RS-content parent are indicated by “+”, and those with heterozygous genotypes are indicated by “H”.

## Data Availability

The data sets supporting the conclusions of this article are available in the China National GeneBank Database (CNGBdb) at https://db.cngb.org/, with the following accession numbers: CNP0008416.

## Abbreviations

The following abbreviations are used in this manuscript:

ARMS-PCR	Amplification refractory mutation system polymerase chain reaction
RS	Resistant starch
GBSSI	Granule-bound starch synthase I
GT	Gelatinization temperature
ECQ	Eating and cooking quality
FMs	Functional markers
MAS	Marker-assisted selection
AAC	Apparent amylose content
LD	Linkage Disequilibrium
